# The Food for Infants

**Published:** 1872-10

**Authors:** 


### THE FOOD OF INFANTS.

Dr. C. A. Coudereau expresses himself in opposition to the generally received opinion that the milk of a wet-nurse is the best substitute for that of the mother when the latter cannot be obtained. He has found in the milk of many wet-nurses, dependent on their want of cleanliness, a peculiar fungus, which will develop under favorable circumstances in every other kind of milk, giving to such milk a peculiar odor, and discoverable in the evacuations of the child. In regard to artificial food, he rejects also beef-tea, as well as Liebig's extract of meat, but recommends a fluid into the composition of which eggs enter largely. He considers that a very nourishing and wholesome kind of drink can be obtained from eight eggs, white and yolk together, beaten up with about two ounces of sugar and enough water to make a pint and a half of fluid. To this he adds a small quantity of lime-water, sulphate of potash, and chloride of sodium. With a fluid so composed he has obtained excellent results.—*Wiener Medizinischen Wochenschrift.*

